# Artificial Protein Coronas Enable Controlled Interaction with Corneal Epithelial Cells: New Opportunities for Ocular Drug Delivery

**DOI:** 10.3390/pharmaceutics13060867

**Published:** 2021-06-12

**Authors:** Carlo Astarita, Sara Palchetti, Mina Massaro-Giordano, Marina Di Domenico, Francesco Petrillo, Silvia Boffo, Giulio Caracciolo, Antonio Giordano

**Affiliations:** 1 Sbarro Institute for Cancer Research and Molecular Medicine, Department of Biology, College of Science and Technology, Temple University, Philadelphia, PA 19122, USA; mina@pennmedicine.upenn.edu (M.M.-G.); marina.didomenico@unicampania.it (M.D.D.); tue91800@temple.edu (S.B.); president@shro.org (A.G.); 2 Department of Molecular Medicine, Sapienza University of Rome, Viale Regina Elena 291, 00161 Rome, Italy; sara.palchetti@uniroma1.it; 3 Department of Ophthalmology, Scheie Eye Institute, Perelman School of Medicine, University of Pennsylvania, Philadelphia, PA 19104, USA; 4 Department of Precision Medicine, University of Campania Luigi Vanvitelli, 80138 Naples, Italy; 5 Department of Ophthalmology, A. O. U. Policlinic-Vittorio Emanuele, 95124 Catania, Italy; francescopetrillo09@gmail.com; 6 Department of Medical Biotechnologies, University of Siena, 53100 Siena, Italy

**Keywords:** ocular drug delivery, protein corona, ocular surface

## Abstract

Topical administration is the most convenient route for ocular drug delivery, but only a minor fraction is retained in the precorneal pocket. To overcome this limitation, numerous drug delivery systems (DDS) have been developed. The protein corona (PC) is the layer of biomolecules (e.g., proteins, sugars, lipids, etc.) that forms around DDS in physiological environments by non-covalent interaction. The PC changes the DDS physical–chemical properties, providing them with a completely novel biological identity. The specific involvement of PC in ocular drug delivery has not been addressed so far. To fulfill this gap, here we explored the interaction between a library of four cationic liposome-DNA complexes (lipoplexes) and mucin (MUC), one of the main components of the tear film. We demonstrate that MUC binds to the lipoplex surface shifting both their size and surface charge and reducing their absorption by primary corneal epithelial cells. To surpass such restrictions, we coated lipoplexes with two different artificial PCs made of Fibronectin (FBN) and Val-Gly-Asp (VGA) tripeptide that are recognized by receptors expressed on the ocular surface. Both these functionalizations remarkedly boosted internalization in corneal epithelial cells with respect to pristine (i.e., uncoated) lipoplexes. This opens the gateway for the exploitation of artificial protein corona in targeted ocular delivery, which will significantly influence the development of novel nanomaterials.

## 1. Introduction

Loading poorly water soluble, poorly permeable and instable drugs into drug delivery systems (DDS) has long been the most efficient way to increase their bioavailability [1]. In the last decades, nanotechnology has continuously offered new solutions for drug delivery such as the possibility to reduce toxicity and increase the therapeutic index of conventional parent drugs. Since 1995, almost 50 nano-pharmaceuticals have received approval by the Food and Drug Administration (FDA) and are currently available for clinical use [2]. Among them, liposomes are the most successful DDS with a dozen approved drug products and many more in clinical development [3,4]. In recent years, liposomes have also emerged as ideal platforms for targeted drug delivery [5]. This strategy requires the surface functionalization of liposomes with ligands such as proteins, antibodies (Abs) and other biomolecules to promote the entry of therapeutics by cognate receptors (over)expressed at the plasma membrane of the target cell [6]. Despite continuous efforts, no targeted liposomal formulation has been approved for clinical practice. Recent research elucidated the reason of the wide gap existing between benchtop discoveries and clinical application of targeted liposomal formulations. It has been clarified that, when dispersed in bodily fluid (i.e., blood, serum, saliva, etc.), liposomes are rapidly covered by biomolecules, forming a shell commonly named as protein corona (PC) that effectively screens the bare liposome surface [7,8]. Recently, the concept is emerging that PC is the biologically relevant interface “seen” and processed by the cell machinery and not the functionalized liposome surface [8,9,10,11,12,13,14]. In topical corneal drug delivery, liposomes come into contact with the tear film, a thin fluid layer composed of water, electrolytes, proteins, lipids, and mucins (MUC). In particular, MUC are a class of highly negative glycosylated proteins with a relevant, but still undefined, role in drug delivery [15]. We therefore have reason to speculate that the interaction between lipids and MUC in the eye may lead to formation of a MUC-enriched PC around liposomes that impairs their uptake by corneal epithelial cells. To test this hypothesis, we incubated a library of four cationic liposome (CL)-DNA complexes (lipoplexes) with MUC and exposed MUC-coated lipoplexes to human primary corneal epithelial cells. The employment of lipoplexes as a model system of ocular DDS seemed to be a logical choice, as they have been recently employed for safe and reliable topical treatment of several ocular diseases [16].

Recent research has also shown that pre-coating DDS with artificial PC can enhance the internalization within target cells [17,18,19,20]. Several artificial coronas are stable upon exposure to body fluid, thus keeping stealth properties and controlling cellular interactions of DDS in physiological environments. As a second goal of this work, we therefore explored the possibility to exploit artificial coronas to enhance cellular uptake of lipoplexes by human primary corneal epithelial cells. To this end, we coated lipoplexes with (i) Fibronectin (FBN) and (ii) Val-Gly-Asp (VGA) tripeptide. These ligands are specifically recognized by receptors and transporters expressed on the ocular surface (i.e., A_v_β5 and PEP-1, respectively). It is worth mentioning that both the artificial coronas could enhance cellular uptake of lipoplexes within human primary corneal epithelial cells. These results are the proof of concept, that coating DDS with artificial PC may be an innovative strategy for efficient corneal drug delivery.

## 2. Materials and Methods

### 2.1. Chemicals

Mucins (Ref. 499643) were purchased from Sigma Aldrich (St. Louis, MO, USA), fibronectin (Ref. 1918-FN) was purchased from R&D system (Minneapolis, MN, USA) and the tripeptide (Val-Gly-Asp) was designed and purchased from GenScript (Piscataway, NJ, USA).

### 2.2. Cationic Liposomes, Lipoplexes and Artificial Protein Corona

Cationic 1,2-dioleoyl-3-trimethylammonium-propane (DOTAP) and (3b-[*N*-(N0, N0 -dimethylaminoethane)-carbamoyl])-cholesterol (DC-Chol), and the zwitterionic helper lipid dioleoylphosphatidylethanolamine (DOPE) were purchased from Avanti Polar Lipids (Alabaster, AL, USA) and used without further purification. Cholesterol was purchased from Sigma-Aldrich. Cationic liposomes (CL) were prepared following standard protocols [21,22]. DC-Chol-Chol (1:1 molar ratio; CL1), DC-Chol-DOPE (1:1 molar ratio; CL2), DOTAP-Chol (1:1 molar ratio; CL3) and DOTAP-DOPE (1:1 molar ratio; CL4) were prepared by dissolving lipids in chloroform, and the solvent was evaporated under vacuum for 2 h. The obtained lipid film was hydrated with ultrapure water to achieve the desired lipid concentration. The obtained liposome solutions were extruded 20 times through a 0.1 µm polycarbonate carbonate filter by the Avanti Mini-Extruder (Avanti Polar Lipids, Alabaster, AL, USA).

To generate the fluorescent labeled lipoplexes (LPX), liposomes were incubated with a fluorescent DNA probe, Label IT plasmid delivery control cy3 (Mirus Bio, Madison, WI, USA), at weight ratio 1:20 for 25 min at room temperature. This value was chosen according to previous findings, as it corresponds to the minimum amount of cationic lipid needed to encapsulate the gene payload and keep the net charge of the complex positive. Finally, biocoronated lipoplexes (or artificial protein corona lipoplexes) were generated by incubating pristine lipoplexes with MUC, FBN and VGA for 1-h at 37 °C, creating 16 different combinations (Table 1).

### 2.3. Size and Zeta-Potential Experiments

The size and zeta-potential distributions of bare lipoplexes and lipoplexes incubated with different biomolecules were measured by a NanoZetasizer apparatus (Malvern, UK). To form lipoplexes, all liposomes were incubated with fluorescent plasmid DNA, and lipoplexes were then incubated with MUC, FBN and VGA as described above. For size and zeta-potential analysis, all the complexes were diluted 1:100 with ultrapure water. Results are given as mean ± standard deviation of five independent replicates.

### 2.4. Cell Culture

Primary corneal epithelial cells (ATCC^®^ PCS-700-010™) were purchased from ATCC (Manassas, VA, USA). The Corneal Epithelial Cell Basal Medium (ATCC^®^ PCS-700-030™) was purchased from ATCC and used according to the manufacturer’s instructions along with the Corneal Epithelial Cell Growth Kit (ATCC^®^ PCS-700-040™). Cells were maintained in in a humidified atmosphere of 5% CO_2_ to retain the proliferative conditions.

### 2.5. Flow Cytometry Experiments

For flow cytometry experiments, 150,000 cells were seeded in 6-well plates. Biocoronated lipoplexes were prepared for incubating pristine lipoplexes with mucin for 1-h at 37 °C. After 24 h, cells were treated with (i) pristine-LPX and (ii) MUC-biocoronated LPX for 30 min, 60 min and 90 min to evaluate cellular uptake. Secondarily, to mimic ocular administration and test the artificial corona, cells were treated with (i) pristine-LPX, (ii) FBN-biocoronated LPX, and (iii) VGA-biocoronated LPX, for 30 min, then washed with phosphate-buffered saline (PBS) and incubated with complete medium for 1 h. After those treatments, cells were washed three times in phosphate-buffered saline (PBS) and harvested with 0.25% trypsin solution (Invitrogen, Sweden). Then, cells were centrifuged 5 min at 1200 rpm, and the pellet was dissolved in PBS (500 μL). The uptake of the different fluorescently labelled lipoplexes was evaluated by 635 nm laser excitation (filter 655−730 nm). Samples were analyzed by flow cytometry (Accuri C6 Flow Cytometer, Accuri Bioscience, San Jose, CA, USA). For all the tested samples, *N* = 3 independent experiments were conducted, each in triplicate. A fixed number of 1.5 × 104 cells was acquired for each sample.

### 2.6. Fluorescence

Primary corneal epithelial cells were grown on Nunc™ Lab-Tek™ II Chamber Slide™ System (8-well Chamber Slide), 10.000 cells for each well for 24 h and then incubated with (i) pristine-LPX and (ii) MUC-biocoronated LPX for 1 h. Fluorescence was performed using a fluorescence Application Kit according to the manufacturer’s protocol (Cell Signaling Technology, Danvers, MA, USA). The coverslips were then rinsed with PBS, and the preparations were mounted with a Vectashield mounting medium (Vector Laboratories, Burlingame, CA, USA). The images were captured and evaluated with an Olympus IX81 deconvolution fluorescence microscope (Olympus Microscopes, Tokyo, Japan). 

### 2.7. Statistical Analysis

Statistical differences were determined by Student’s *t* test. *p* < 0.05 was considered significant (*) and *p* < 0.001 was consider strongly significant (**).

## 3. Results

### 3.1. Effect of Biomolecular Corona on Size and Zeta-Potential of Lipoplexes

**Figure 1:**
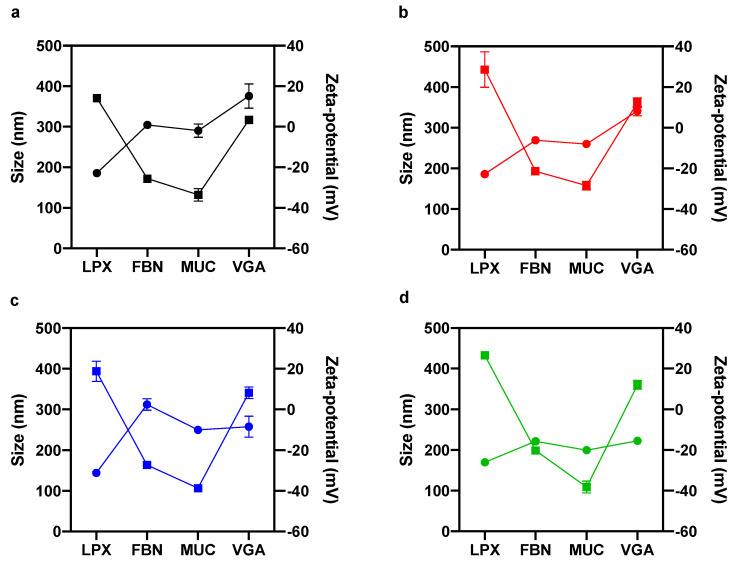
Size (circles) and zeta-potential (squares) of lipoplexes (LPX) before and after interaction with Fibronectin (FBN), mucin (MUC) and Val-Gly-Asp (VGA) tripeptide: LPX1(**a**); LPX2 (**b**); LPX3 (**c**); LPX4 (**d**). Results are given as mean ± standard deviation of *N* = 5 independent replicates.

As size and zeta-potential are two key factors affecting cellular uptake of exogeneous vesicles, these two features of lipoplexes were assessed first. Pristine lipoplexes (i.e., in the absence of the artificial corona) were smaller than 200 nm in size (Figure 1, circles) and positively charged (Figure 1, squares) for all four formulations: LPX1, LPX2, LPX3 and LPX4. According to previous literature, the latter finding can be explained assuming that the positively charged lipid surface of lipoplexes was only in part decorated by negatively charged DNA molecules. Incubation with FBN, MUC and VGA affected both size and surface charge of lipoplexes. In the presence of FBN-, MUC- and VGA-artificial PC, the size of lipoplexes increased with values fluctuating between ~200 nm (MUC-biocoronated LPX4) (Figure 1d, circles) and ~380 nm (VGA-biocoronated LPX2) (Figure 1b, circles). In principle, some interactions between DNA and proteins could take place at the particle surface, but exploring this aspect goes beyond the scope of the physical–chemical characterization, which is aimed at determining the size and zeta-potential of the biocoronated complexes emerging from the interaction between lipoplexes and proteins. Clear effects for zeta potential of biocoronated lipoplexes were also found. Adsorption of FBN and MUC reversed the zeta-potential from positive to negative values. The zeta-potential of the VGA-coated lipoplexes was instead still positive, but lower than that of pristine lipoplexes (Figure 1a–d squares).

### 3.2. Inhibitory Effect of Mucin-Enriched Biomolecular Corona on Cellular Uptake

Tear film is composed of three components (i.e., lipids, aqueous and mucus), the volume is 6–8 µL and the turnover is approximately 16% min. of the total volume, and the rate increases after drug instillation because of reflex tearing, inducing an accelerated drainage [23] The innermost component contains mainly MUC that are secreted or membrane-associated [24]. To mimic physiological conditions, fluorescently labelled pristine LPX and MUC-biocoronated LPX were incubated with primary corneal epithelial cells for 30 min, 60 min and 90 min. As a first step, cellular uptake was explored by fluorescence imaging.

**Figure 2:**
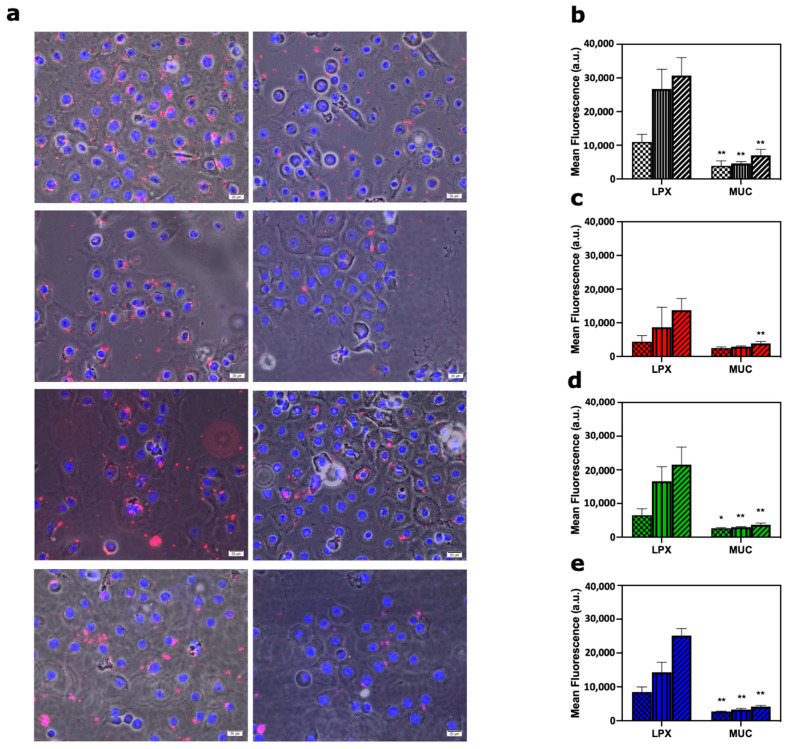
Representative images for subcellular localization of fluorescently labelled (red) pristine-LPX and MUC-biocoronated lipoplexes in primary corneal epithelial cells after 60 min treatment. Cell nuclei were stained with DAPI. Scale bars are 20 microns. (**a**) Mean fluorescence FACS analysis acquired by 635 nm laser excitation (filter 655–730 nm) was performed to assess the uptake by primary corneal epithelial cells following treatment with Pristine LPX and MUC-biocoronated LPX at three incubation times (30, 60 and 90 min). LPX1(**b**); LPX2 (**c**); LPX3 (**d**); LPX4 (**e**). Results are given as average of *N* = 3 independent measurements ± standard deviation. Statistical differences were determined by Student’s *t* test: *p* < 0.05 was considered significant (*) and *p* < 0.001 was consider strongly significant (**).

Representative fluorescence staining images collected 60 min after incubation of lipoplexes with primary corneal epithelial cells (Figure 2a; Figure S1). Next, internalization of both pristine and MUC-biocoronated lipoplexes was quantified by flow cytometry. Flow cytometry results were in full agreement with confocal imaging, indicating that adsorption of MUC at the lipoplex surface strongly inhibited cellular uptakes at all the time points (30, 60 and 90 min) and for all the lipoplexes formulations (Figure 2b–e).

### 3.3. Enhanced Cellular Uptake of Lipoplexes by Artificial Protein Corona

**Figure 3:**
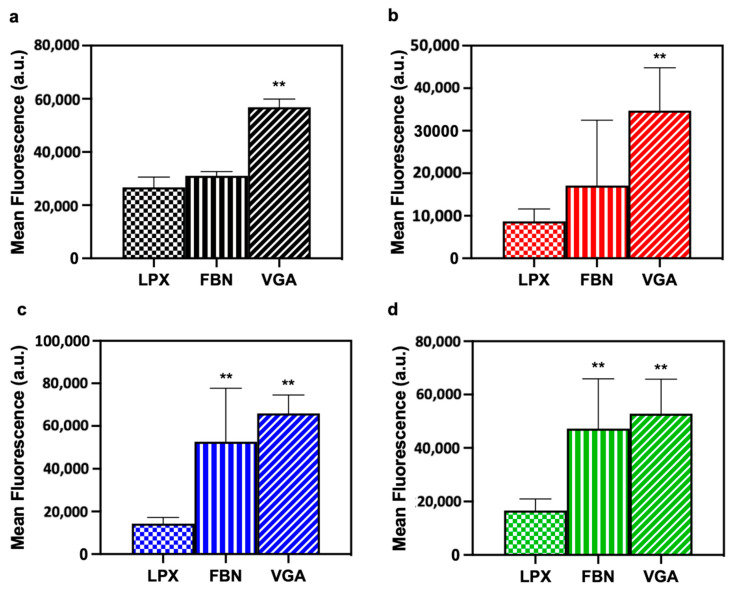
Mean fluorescence acquired by 635 nm laser excitation (filter 655–730 nm) was performed by FACS analysis to assess the uptake by primary corneal epithelial cells following treatment with pristine lipoplexes (LPX), FBN-biocoronated lipoplexes and VGA-biocoronated lipoplexes. LPX1(**a**); LPX2 (**b**); LPX3 (**c**); LPX4 (**d**). Results are given as average of *N* = 3 independent measurements ± standard deviation. Statistical differences were determined by Student’s *t* test: *p* < 0.001 was consider strongly significant (**).

Considering the challenges in ocular drug delivery, several approaches are needed to have an optimal ophthalmic formulation in a way to increase ocular surface drug absorption. We assessed whether artificial PC may enhance cellular uptake by primary corneal epithelial cells. To this end, a logical choice was the employment of FBN and VGA because they are specifically recognized by extracellular receptors of primary corneal epithelial cells. Cellular uptake of biocoronated lipoplexes was analyzed through flow cytometry and pristine lipoplexes (i.e., in the absence of PC) were used as a control. The biocoronated lipoplexes (both FBN and VGA) are avidly internalized by primary corneal epithelial cells if compared to all pristine LPX (Figure 3a–d; Figure S2).

## 4. Discussion

Liposomes are a versatile DDS with numerous applications ranging from chemotherapeutics to gene therapy and ocular delivery. However, despite continuous efforts and clinical trials, only a limited number of liposomal formulations were approved by regulatory authorities. After a decade of intense research [25], we now believe that the gap existing between lab discoveries and routine clinical practice is likely due to our limited knowledge of the biological identity assumed by liposomes under physiological conditions. Indeed, when liposomes come into contact with bodily fluids (e.g., the blood, interstitial fluids, urine, etc.), they are coated by a complex PC that rapidly becomes the biological interface “seen” by the biological systems. As the primary particle surface remains buried and no longer accessible, the liposome-PC is the main factor shaping liposome–cell interactions and the cell response [26]. To date, the role of PC on ocular drug delivery has been poorly addressed [27]. The trans-corneal route provides the greatest absorption for most of the clinically approved drugs and the superficial layer of the cornea is the epithelium that represents a great barrier for paracellular diffusion of pharmaceuticals [28]. The use of mucoadhesive substances with the purpose of increasing drug absorption has been the subject of several investigations [29]. However, poor attention has been given to the fact that mucines are not only at the surface of the corneal epithelial cells, but also dispersed in the tear film, exerting a protective function over the ocular surface and displaying the importance of precorneal tear film as a significant barrier to drug uptake [24]. According to above stated considerations, we put forth the concept that interaction between liposomes and tear film components may change the synthetic properties of liposomes, providing them with a totally new biological identity. To fulfill this gap and test our suggestion about the role of PC on corneal drug delivery, we explored the interaction between MUC and a library of four lipoplexes with different lipid compositions (Table 1). We demonstrated that exposing lipoplexes to a MUC-enriched medium led to formation of MUC-coated lipoplexes with different sizes and surface charges with respect to their pristine counterpart (Figure 1). In particular, adsorption of MUC at the lipoplex surface reverses the zeta-potential from positive to negative values. This is worthy of note as it has been demonstrated that the association of lipoplexes with mammalian cells is controlled by non-specific electrostatic interactions [22]. In more detail, cationic lipids favorably interact with cell surface proteoglycans, thus promoting cellular association and internalization [30]. By fluorescence staining, we were able to show that pristine-LPX were internalized in the cellular compartment whereas MUC-biocoronated LPX were only in the cellular surroundings (Figure 2a). Moreover, our results suggest that MUC bind to cationic lipids thus impairing lipoplex–cell interaction and sensibly decreasing particle internalization within primary corneal epithelial cells (Figure 2b–e). Next, we asked whether creating an artificial corona at the lipoplex surface could promote favorable interaction with primary corneal epithelial cells. Several previous works demonstrated that corona components control interaction with target cells. For instance, Caracciolo et al. [31] demonstrated that a Vitronectin (VTN)-enriched PC could boost internalization of lipoplexes within MDA-MB-435S breast cancer cells that express high levels of the VTN-receptor (i.e., A_v_β_3_ integrin). In subsequent studies [32,33], it has been shown that apolipoprotein-enriched coronas can promote association of nanoparticles to specific lipoprotein receptors, i.e., low-density lipoprotein receptor (LDLR) and scavenger receptor class B, type I (SRB1) that are (over)expressed in several conditions (e.g., renal cell carcinoma, hepatocellular carcinoma, lymphoma, melanoma, etc.).

The corneal epithelium is the major target for ocular drug delivery; thus, overexpressed receptors and transporters such as PEP-1 [34], A_v_β_5_ [35]_,_ LAT-1 [36], ASCT1 [37] and OATP [38] are investigated as targets. However, besides chitosan and Hyaluronic Acid (HA) coated liposomes as ocular delivery system, no other investigations have been carried out to date [39,40]. To test this suggestion, we fabricated two variants of artificial corona (i.e., FBN- and VGA-biocoronated lipoplexes, Table 1) to target Avβ_5_ receptor and PEP-1 receptor, respectively. Our results showed that both artificial coronas were able to promote massive cellular uptake of lipoplexes by primary corneal epithelial cells. We emphasize that cellular uptake of biocoronated lipoplexes was significantly higher that than that of the pristine ones (Figure 3a–d), possibly switching the uptake mechanism from a non-specific and electrostatic-mediated mechanism for pristine lipoplexes to receptor-mediated internalization for biocoronated ones, although this is not yet proven with this paper.

## 5. Conclusions

**Figure 4:**
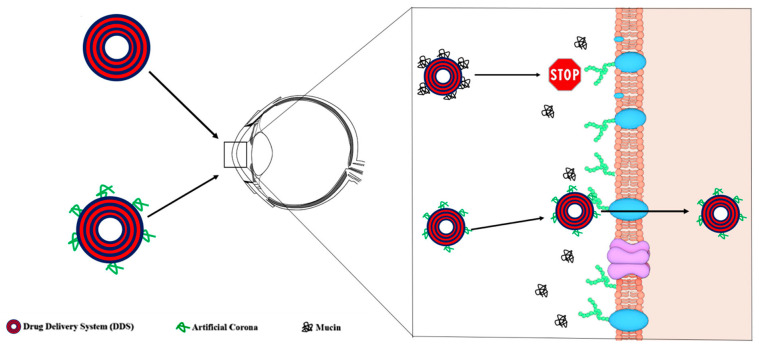
Upon topical eye administration drug delivery systems are coated by mucins that inhibit association with corneal epithelial cells. On the other side, artificial coronas with affinity for cell receptors promote massive internalization by a receptor-mediated mechanism.

Figure 4 summarizes the main conclusion of this work. First, we have demonstrated that interaction with MUC changes the physical–chemical properties of lipoplexes and provide them with a totally new biological identity. In particular, the inversion of the lipoplexes surface charge is likely to reduce the electrostatic interactions within corneal epithelial cells; moreover, the MUC layer at the particle surface was responsible for inhibition of cellular internalization, thus shedding light on the possible inhibitory role of mucins for ocular drugs absorption, which is still not clear. To overcome such limitations, we have successfully exploited the fabrication and use of artificial coronas to target receptors of corneal epithelial cells in order to boost cellular uptake. This work has therefore provided the proof of concept that coating nanocarriers with artificial coronas with a natural affinity for receptors of corneal cells is a promising strategy for enhanced topical ocular drug delivery.

This formulation has several advantages, is highly reproducible, cost efficient and versatile since several other biomolecules might be tested to evaluate their uptakes efficiency. We believe that the exploitation of artificial protein corona in targeted ocular delivery will significantly influence the development of novel nanomaterials.

## Figures and Tables

**Table 1:** Schematic representation of the obtained liposome, pristine lipoplexes (LPX) and biocoronated LPXs.

Liposome	Fluorescent DNA	Fluorescent DNA + Mucin	Fluorescent DNA + Fibronectin	Fluorescent DNA + Val-Gly Asp
CL1	Pristine LPX1	MUC-biocoronated LPX1	FBN-biocoronated LPX1	VGA-biocoronated LPX1
CL2	Pristine LPX2	MUC-biocoronated LPX2	FBN-biocoronated LPX2	VGA-biocoronated LPX2
CL3	Pristine LPX3	MUC-biocoronated LPX3	FBN-biocoronated LPX3	VGA-biocoronated LPX3
CL4	Pristine LPX4	MUC-biocoronated LPX4	FBN-biocoronated LPX4	VGA-biocoronated LPX4

## Data Availability

The data presented in this study are available in Artificial protein coronas enable controlled interaction with corneal epithelial cells: new opportunities for ocular drug delivery.

## Supplementary Materials

The following are available online at https://www.mdpi.com/article/10.3390/pharmaceutics13060867/s1, Figure S1. Representative images for subcellular localization of fluorescently labelled (red) pristine-LPX and MUC-biocoronated lipoplexes in primary corneal epithelial cells after 60 min. treatment. Cell nuclei were stained with DAPI. Results are given as average of *N* = 3 independent measurements ± standard deviation, Figure 2. FACS analysis to assess the uptake by primary corneal epithelial cells following treatment with pristine lipoplexes (LPX), FBN-biocoronated lipoplexes and VGA-biocoronated lipoplexes. Results are given as average of *N* = 3 independent measurements. Fluorescence acquired by 635 nm laser excitation (filter 655–730 nm).
