# Supplementary material for: Artificial Protein Coronas Enable Controlled Interaction with Corneal Epithelial Cells: New Opportunities for Ocular Drug Delivery
Source: Pharmaceutics. 2021 Jun 12;13(6):867. doi: 10.3390/pharmaceutics13060867 (PMC8231102; doi:10.3390/pharmaceutics13060867)
Supplement: Supplementary file 1 [file pharmaceutics-13-00867-s001.zip › pharmaceutics-1034108-supplementary.pdf]

pharmaceutics

Supplementary Materials: Artificial Protein Coronas Enable Controlled Interaction with Corneal Epithelial Cells: New Opportunities for Ocular Drug Delivery

Carlo Astarita , Sara Palchetti, Mina Massaro-Giordano, Marina Di Domenico, Francesco Petrillo, Silvia Boffo, Giulio Caracciolo and Antonio Giordano

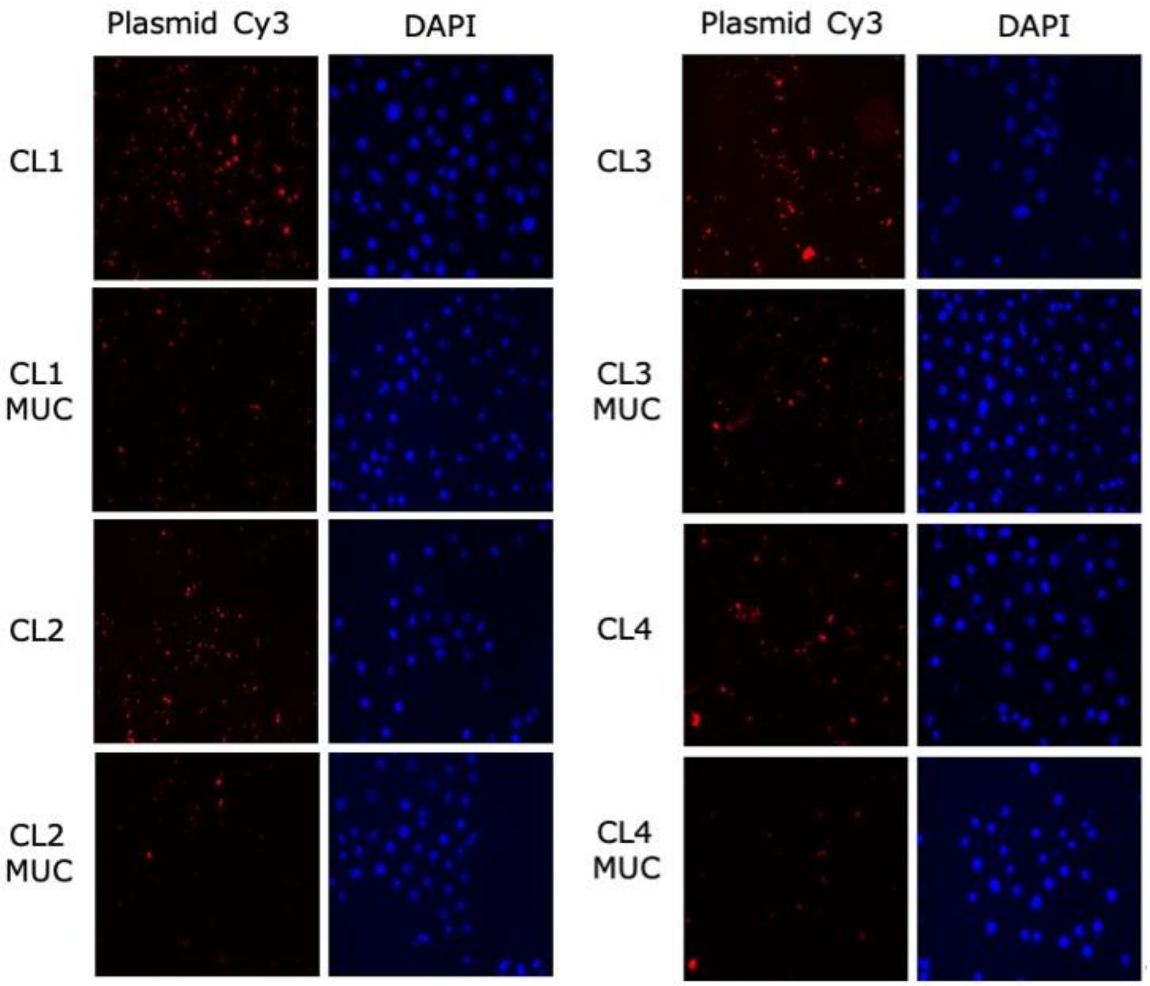

Figure S1. Representative images for subcellular localization of fluorescently labelled (red) pristine-LPX and MUC-biocoronated lipoplexes in primary corneal epithelial cells after 60 min. treatment. Cell nuclei were stained with DAPI. Results are given as average of *N* = 3 independent measurements ± standard deviation.

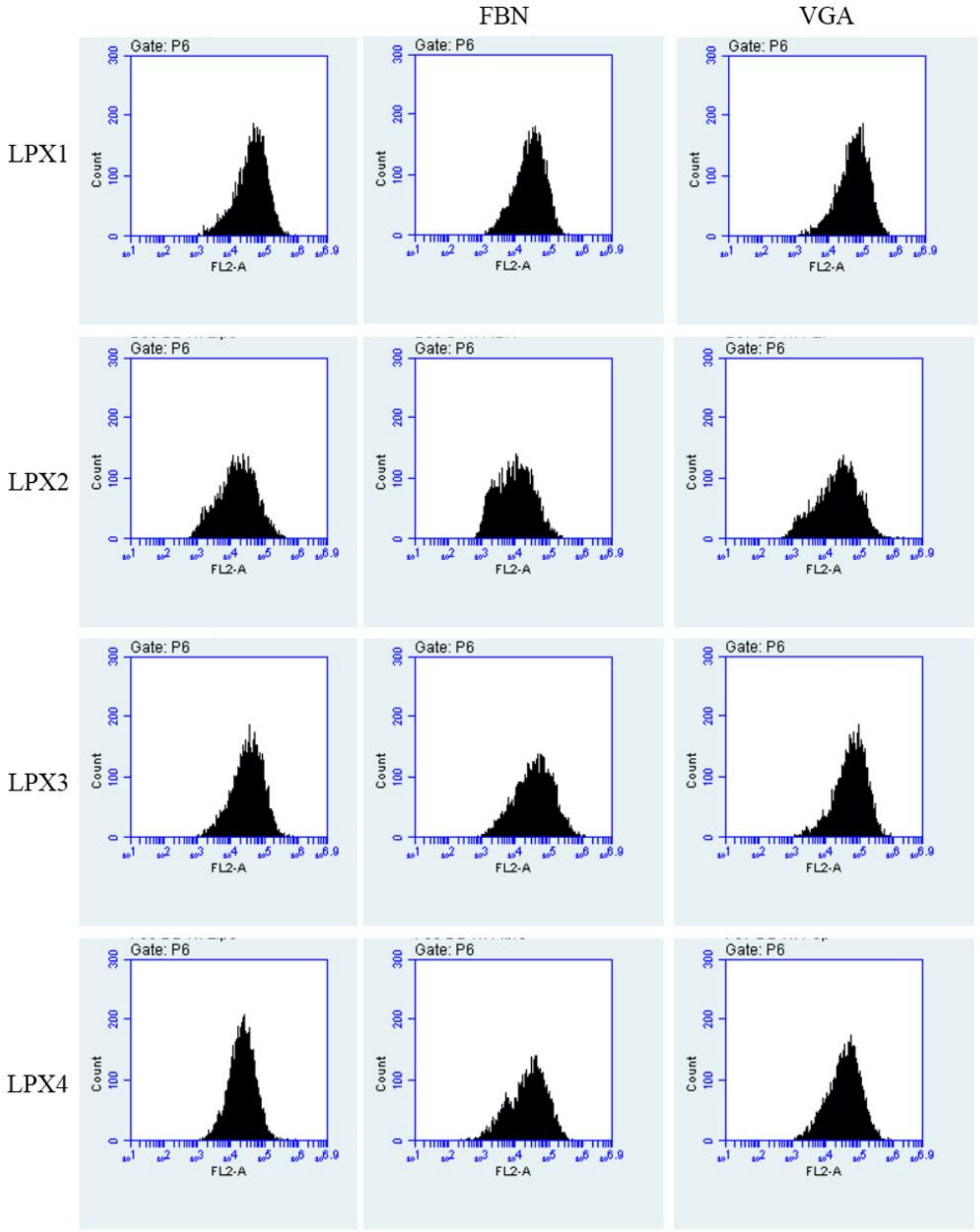

Figure S2. FACS analysis to assess the uptake by primary corneal epithelial cells following treatment with pristine lipoplexes (LPX), FBN-biocoronated lipoplexes and VGA-biocoronated lipoplexes. Results are given as average of $N = 3$ independent measurements. Fluorescence acquired by 635nm laser excitation (filter 655–730nm).
